# Postponement of the Newborn Hearing Screening during the COVID-19 Pandemic; Parental Experiences and Worries

**DOI:** 10.3390/ijns10010026

**Published:** 2024-03-21

**Authors:** Rosanne B. van der Zee, Sanne L. Peet, Noëlle N. Uilenburg, Hedwig J. A. van Bakel, Evelien Dirks

**Affiliations:** 1 The Dutch Foundation for the Deaf and Hard of Hearing Child (NSDSK), 1073 GX Amsterdam, The Netherlands; speet@nsdsk.nl (S.L.P.); nuilenburg@nsdsk.nl (N.N.U.); edirks@nsdsk.nl (E.D.); 2 School of Social and Behavioral Sciences, Tranzo, Tilburg University, 5000 LE Tilburg, The Netherlands; h.j.a.vanbakel@tilburguniversity.edu; 3 Department of Psychology, Utrecht University, 3584 CH Utrecht, The Netherlands

**Keywords:** newborn hearing screening, neonatal hearing screening, COVID-19, parental experiences, population screening program, UNHS

## Abstract

Early identification of hearing loss through newborn hearing screening followed by an early start of intervention has proven to be effective in promoting speech and language development in children with hearing loss. During the COVID-19 pandemic, newborn hearing screening was postponed for a group of newborns in the Netherlands. Therefore, meeting the guidelines for early identification was at risk. In this study, we examine parental attitudes, beliefs, and experiences concerning the hearing screening during the COVID-19 pandemic. Our results indicated that parents (*n* = 1053) were very positive about newborn hearing screening and their experiences with the screening, even during the COVID-19 pandemic. Parents’ beliefs on the information provision around newborn hearing screening were more inconsistent. The results showed that parents with a postponed hearing screening felt less informed about the hearing screening than parents without a postponed screening. Furthermore, child and family characteristics affected how parents experienced newborn hearing screening. Parents with a premature child were more worried about the hearing abilities of their child before the screening took place. The results also indicate that deafness in the family might lead to parental worries around newborn hearing screening.

## 1. Introduction

Around 1.23 per 1000 children in the Netherlands are born with bilateral hearing loss [1]. These children are at risk for delays in their speech and language outcomes [2,3]. Early identification of hearing loss through newborn hearing screening followed by early start of intervention has proven to be effective in promoting speech and language development in children with hearing loss [4,5,6]. It is recommended that screening procedures follow the international Joint Committee on Infant Hearing 1-3-6 guidelines [7], which state that all babies should be screened no later than 1 month of age, and those with hearing loss should be identified by 3 months and begin with early intervention no later than 6 months of age. In the Netherlands, this 1-3-6 guideline is followed via the nationwide neonatal hearing screening program [8].

Children who meet these 1-3-6 guidelines have better language outcomes than those who do not [4,6,9,10]. It is thus important to ensure that all newborns with hearing loss meet these guidelines. In this paper, the focus lies on the newborn hearing screening, the first step in achieving the 1-3-6 guidelines. During the COVID-19 pandemic, achieving these guidelines was at risk due to postponement of the hearing screening for a group of newborns. The main aim of this study is to examine parental attitudes, beliefs, and experiences concerning the hearing screening in the Netherlands during the COVID-19 pandemic. This will provide us with helpful knowledge to make appropriate considerations in case of future pandemic crises. 

### 1.1. Newborn Hearing Screening in the Netherlands

In the Netherlands, all newborns are eligible for free newborn hearing screening, which is carried out by the nationwide youth healthcare program. The aim of the newborn hearing screening is to identify children with a permanent hearing loss of at least 40 dB in one or both ears in order to ensure an early start of intervention. If children do not pass the hearing screening, they are referred to a speech and hearing center for an audiological assessment. The newborn hearing screening is voluntary and performed in three stages: For the first screening, the otoacoustic emission (OAE) method is used. If a child does not pass this screening, a second OAE screening follows. If a child does not pass the second OAE screening for one or both ears, a third screening with automated auditory brainstem response (A-ABR) is carried out. In the Netherlands, most children are born at home or in a maternity ward from which they are discharged within a day. Therefore, hearing screening takes place at home for most newborns (approximately 75%), together with the newborn blood spot screening. In some regions of the country, parents need to visit a youth healthcare center with their baby for the hearing screening of their newborn. Children who are admitted to the neonatal intensive care units (NICUs) follow a separate protocol and are screened within the NICU program.

During the COVID-19 pandemic, newborn hearing screening was postponed from 24 March to 4 May 2020. For the 17,690 children that were not screened in this period [11], the hearing screening was postponed and caught up within three months. Due to the postponement, the waiting time for parents was longer. In addition, postponed hearing screenings often took place at the youth healthcare center instead of in the home environment. Even during this first year of the COVID-19 pandemic in 2020, participation rates of the hearing screening were very high, where 99.5% of the newborns participated in the first screening round [11]. However, postponement of the newborn hearing screening did have a large impact on the timeliness of screening, diagnosis, and intervention; the 1-3-6 guidelines. The first OAE screening was on time for only 89.4% of the children in 2020, compared to 99.3% in 2019 [11,12]. This resulted in a diagnosis before the age of 3 months for 82.6% of the children in 2020, compared to 93.5% of the children in 2019.

There are several explanations for the success rate of the Dutch newborn hearing screening program. Newborn hearing screening was implemented nationwide in the Netherlands in 2006. The Dutch Institute for Public Health and the Environment’s Center for Population Screening (RIVM-CvB) directs the screening program on behalf of the Dutch Ministry of Health, Welfare, and Sports. The implementation of screening programs needs to meet the public values as described by the government: quality, accessibility, and affordability. National guidelines and quality requirements on newborn hearing screening are defined in a procedure manual [8]. In the Netherlands, hearing screening is accessible for free for all newborns. Hearing screening takes place mostly in the home environment or in a nearby youth healthcare center. In addition, the quality of the newborn hearing screening program is regularly monitored, regionally by the youth healthcare organizations as well as nationally by The Netherlands Organization for Applied Scientific Research (TNO) at the commission of the National Institute for Public Health and the Environment, Center for Population Screening Program (RIVM-CvB). Outcomes and quality of the program are discussed regularly between all involved parties. Furthermore, all screeners in the Netherlands follow the same training program for newborn hearing screening in which considerable attention is paid to conversing with parents. Parents are already informed about the newborn hearing screening during pregnancy by maternity caregivers. Positive parental attitudes on newborn hearing screening in general and the received information about the screening beforehand could contribute to high participation rates [13].

### 1.2. Parental Experiences, Beliefs, and Worries

Parents and caregivers are important stakeholders in the successful execution of newborn hearing screening programs [14]. A recent systematic review on the acceptability of childhood screening, including 13 studies on newborn hearing screening, indicated that parental affective attitudes and their understanding of the screening were important constructs to consider, when aiming to maximize participation rates [13]. Positive attitudes toward newborn hearing screening have been reported in studies addressing parents’ feelings, perceptions, and experiences [15,16,17,18]. Most parents consider the screening to be worthy [19]. Overall satisfaction levels concerning the newborn hearing screening are generally high [18,19,20,21] and increase when expecting mothers receive educational information regarding the screening during their pregnancy [20]. Parents report higher overall satisfaction and higher satisfaction with the screener and the procedure when they receive information beforehand. Information provision during pregnancy seems thus an important factor. Additionally, information provision by the screener during the screening procedure itself also contributes to parents’ satisfaction. In a study during the implementation of the Dutch newborn hearing screening [22], 623 parents were questioned about the information provision in various screening rounds. Almost 90% of these parents reported that the information provision by the screener was sufficient and around 80% of the parents were satisfied with the information by the screener. Parental experiences with the newborn hearing screening are also affected by factors such as professional communication and manner of the screener [23]. In this implementation study in the United Kingdom, many parents commented on the value they placed on how “kind”, “patient”, and “nice” screeners were. The authors concluded that parents’ reflections on the screeners were not just about what they said, but more about their personality and character. 

Overall, findings seem to indicate that parents have positive attitudes toward newborn hearing screening and that they are satisfied with the received information. However, their experiences and worries may be affected by specific child and family factors. For example, when parents already have other children who passed the newborn hearing screening, they may have more prior knowledge and less worries. Furthermore, if children have medical concerns, when born prematurely for example, parents may experience the screening differently and may have more worries. This could also account for parents with deafness in the family, for example, when a parent or another child in the family has a hearing loss. In a narrative interview study of parents with deaf children, two families noted that they were worried about the outcome of the screening because deafness already existed in the family [23]. Newborn hearing screening during the COVID-19 pandemic might entail its own type of worries around the screening and hearing loss.

### 1.3. Current Study

The main aim of the current study is to gain insight on parental experiences and worries around the newborn hearing screening during the COVID-19 pandemic. The following research questions are addressed:

1. How did parents experience the hearing screening of their newborn during the first COVID-19 lockdown?
2. Are there differences in parents’ worries and experiences concerning the hearing screening of their newborn in case of a postponed hearing screening compared to a timely hearing screening during the first COVID-19 lockdown?
3. To what extent do child and family characteristics affect parents’ worries and experiences of the hearing screening during the first COVID-19 lockdown?

## 2. Materials and Methods

### 2.1. Participants

The sample of the present study is part of a longitudinal study (Baby2020, van Bakel and Dirks, Tilburg University and NSDSK). The study started in March 2020 to investigate the influences of COVID-19 (restrictions) on parenthood and child development. During the first wave, the study focused on the impact of the postponement of the newborn hearing screening program during the first COVID-19 lock down in the Netherlands. The link to the online questionnaire was distributed through social media (Facebook and LinkedIn) and by flyers at youth healthcare centers in the Netherlands. This resulted in a sample spread across the whole country. As inclusion criteria, (1) children had to be born between March and May 2020 and (2) parents had to understand Dutch in order to fill in the questionnaire. Before starting the online questionnaire, participants gave informed consent. As a reward for participation, parents could win an Instax camera. This project was approved by the Ethics Committee of the University of Tilburg (ID number RP186). In wave 1 of the study, a total of 1053 parents participated.

The questionnaire was filled in by 1017 (96.6%) mothers and 36 (3.4%) fathers. Their children were between 1 and 29 weeks old (*M* = 11.11, *SD* = 5.06) at the time they filled in the questionnaire. In the total sample, 532 (50.5%) children were boys and 521 (49.5%) were girls. Parents were between 16 and 50 (*M* = 31.72, *SD* = 4.15) years old at the time of answering the questions. Parents’ educational level varied from no education to post-graduate, with 66.6% in higher education or above. Other participant characteristics such as birth order, prematurity, and hearing loss in the family can be found in Table 1. 

### 2.2. Measures

In an online questionnaire, parents answered questions on the hearing screening, such as whether the screening had taken place (at home or elsewhere) or whether the screening was postponed because of the COVID-19 restrictions. Participants also reported on the outcomes of the first hearing screening and, if applicable, on the second and third hearing screening. In addition, parents rated 12 statements to evaluate their beliefs, experiences, and worries about the hearing screening on a scale from ‘Completely disagree’ (1) to ‘Completely agree’ (5). Example statements are ‘I received enough information on the procedures of the hearing screening’ and ‘I felt relieved after the hearing screening of my baby’. These statements were based on an existing questionnaire which was used during the implementation study of newborn hearing screening in The Netherlands [22].

### 2.3. Analyses

Chi-squared tests of independence were performed to examine whether parents’ thoughts on the hearing screening differed based on three characteristics. These characteristics were (1) whether the hearing screening was postponed or not, (2) whether the child was born premature or not, and (3) whether this was the parents’ first child or not. Parents’ thoughts on the hearing screening were measured with (1) three statements about their opinion on the information they received and (2) three statements about their worries. This resulted in a total of 18 chi-squared tests. There were no missing data and no outliers identified. To decrease the false discovery rate due to multiple testing, the Benjamini–Hochberg correction was used [24]. After controlling for multiple testing with the Benjamini–Hochberg correction, all 18 chi-squared statistics remained significant with a false discovery rate of 0.05. Significance of all tests were investigated using a significance level of 0.05. 

## 3. Results

A total of 1053 parents reported on the outcomes of the first hearing screening. After the first hearing screening, 37 children (3.5%) did not pass the first screening and needed a second OAE screening. Of those, 23 children (2.2%) needed a third AABR hearing screening as well. After these screenings, five children (0.5%) were referred to a speech and hearing center for further investigation of possible hearing loss. In Table 2, the outcomes of 12 statements on the hearing screening as reported by parents are presented. The beliefs on and experiences with the hearing screening are positively evaluated by most parents. Parents’ report on the information they received around the hearing screening is more divided. Most parents did not report any worries about the hearing abilities of their child. However, some parents did not feel relieved (6.4%) and some parents were still worried about the hearing abilities of their child after the screening (1.0%). 

To examine if the postponement of the hearing screening during the COVID-19 lockdown resulted in different experiences and worries among parents, we compared the parental experiences and worries of a group of newborns that received a postponed hearing screening to a group of newborns that did not. In addition, the influence of child characteristics such as birth order and prematurity on parents’ worries and experiences were examined. In Table 3, the results of 18 chi-squared tests of independence are reported. 

### 3.1. Postponement of Newborn Hearing Screening

We investigated whether postponement of the hearing screening was related to how parents evaluated the information around the screening and the worries they experienced. It turned out that parents in the postponed screening group reported more frequently that they did not receive enough information on the procedures of the hearing screening (Table 3), X^2^ = 11.86, *p* = 0.002, compared to parents without a postponed screening. Furthermore, parents with a postponed screening experienced that the procedure after the hearing screening was less clearly explained to them, X^2^ = 20.36, *p* < 0.001. Regarding the worries of parents, the parents for whom the screening was not postponed reported more often that they were relieved after the screening than parents for whom the screening was postponed. 

### 3.2. Child and Family Characteristics

Child characteristics in relation to parents’ experiences with the hearing screening were investigated in this study as well. First, we investigated whether parents with a prematurely born child experienced the hearing screening in a different manner than parents whose child was not born prematurely. It turned out that parents with a prematurely born child were more often worried about the hearing of their child than parents without a prematurely born child (Table 3), X^2^ = 13.34, *p* = 0.003. Having a prematurely born child or not did not affect parents’ beliefs on information provision and their worries after the hearing screening.

Second, we investigated whether the experiences with the hearing screening differed if the screening concerned parents’ first child or if they already had other children. It turned out that parents for whom it was not their first child were, overall, more positive about the information provision than parents for whom it was their first child. Parents who already had other children more often agreed to have received enough information on the procedures during (X^2^ = 11.53, *p* = 0.003) and after (X^2^ = 13.20, *p* = 0.001) the hearing screening. In addition, they more often reported to have received enough information to decide whether they wanted to participate in the hearing screening, X^2^ = 13.91, *p* < 0.001. No differences were found in the worries of parents whether the screening concerned their first child or not.

Third, we investigated a group of parents that had experience with deafness in the family (*n* = 37, 3.5%). This group was too small to investigate using chi-squared tests of independence. Therefore, only descriptive statistics for this specific group are reported. Based on the hypotheses, we focused on their worries around the hearing abilities of their child around the time of screening. In the period before the screening, 5 of 37 parents (13.5%) were worried about the hearing abilities of their child. Many parents (83.8%) felt relieved after the screening of their child. However, two parents (5.4%) reported to remain worried after the screening. Four of the thirty-seven children (10.8%) needed a second and third screening. 

## 4. Discussion

In this study, we investigated parents’ experiences of the hearing screening of their newborn during the first COVID-19 lockdown. During this lockdown, achieving the 1-3-6- guidelines was at risk. Even in such challenging times, the quality of the screening program was high and parents reflected positively on newborn hearing screening. However, the information provision around newborn hearing screening (during the COVID-19 lockdown) might require more attention. 

Overall, parents were positive about newborn hearing screening and their experiences with the screening. These findings are in line with previous findings during the implementation of the Dutch newborn hearing screening [22] and international satisfaction levels [18,19,20,21]. More than 90% of the parents in our sample would recommend the newborn hearing screening to friends, even though the screening procedure might have been different due to COVID-19 restrictions. 

Parents’ beliefs on the information provision around newborn hearing screening were more inconsistent. Most of the parents were positive about the amount of information they received on the procedure of the screening, which was provided right before the screening took place by one of the screeners. However, only half of the parents believed that the information provision before the screening, on which parents could base their decision to let their baby screen or not, was enough. Parents should receive information on newborn hearing screening from maternity care providers during the last trimester of the pregnancy. In the COVID-19 pandemic, appointments with maternity care providers during pregnancy were reduced and/or replaced by telephone or online consultations [25]. Presumably, this may have impacted the information provision on newborn hearing screening beforehand. However, we are unable to compare results during the pandemic with results before or after the pandemic. Perhaps the information provision to parents needs more attention at all times in order to improve the quality of the Dutch newborn hearing screening program even further. 

### 4.1. The Influence of Postponement of Newborn Hearing Screening on Parents’ Experiences

In the first COVID-19 lockdown, the newborn hearing screening was postponed for several weeks. Our results indicated that parents with a postponed hearing screening felt less informed about the procedures of the hearing screening than parents without a postponed screening. In addition, the procedure after the hearing screening was less clear to parents with a postponed screening. Presumably, postponed hearing screening appointments lasted shorter because screening often took place at a youth healthcare center instead of in the home environment. This may have impacted the information provision from the screeners.

Furthermore, we examined if the relief after the screening differed for parents with and without a postponed screening. It turned out that parents of children whose screening was not postponed felt more relieved after the screening than parents of children with a postponed screening. Newborn hearing screening usually takes place in the first few weeks after birth. Children with a postponed hearing screening were several weeks or already a few months old. When babies are just a little older, parents might be less worried in general or they might have already noticed their child’s reactions to sounds. 

### 4.2. Parents’ Experiences of the Hearing Screening in Relation to Child Characteristics

It was also examined if the parents’ experiences of the hearing screening were related to certain child characteristics. It turned out that, before the screening took place, parents whose baby was born prematurely were more often worried about the hearing abilities of their child than parents whose child was not born prematurely. Premature children, and therefore their parents, can have a more challenging start which might impact the worries that parents experience. After the hearing screening, parents with and without prematurely born children were similar in their worries around the hearing abilities of their children. Only a small amount of parents were still worried after the hearing screening. This seems to indicate that the hearing screening reassured parents on the hearing abilities of their child. Even though parents of prematurely born children may have a lot of information to process in the first weeks after the birth of their child, they did not differ in their experiences with the information provision of the hearing screening compared to other parents. 

Secondly, it was examined whether the experiences of parents for whom it was their first child getting screened differed from parents for whom it was not their first child. Parents who already had other children turned out to be more satisfied about the information provision during and after the screening. This can be explained by the fact that these parents are not solely dependent on the information provision but can also draw on previous experiences with the hearing screening. For parents for whom it is their first child getting screened, the procedures might need more explanation. 

Furthermore, our study indicates that deafness in the family might lead to parental worries around newborn hearing screening, which is in line with previous studies [23]. 

### 4.3. Strengths, Limitations, and Directions for Future Research 

One of the strengths of our study is the high number of participants, especially in such a special year during the COVID-19 pandemic. There were not only many parents willing to participate, but all participants were living throughout the country and had varying levels of education. With this study, we focused on the beliefs and experiences of parents, an important but often neglected aspect of the quality of screening programs. It is unique that we were able to question parents on their experiences during a lockdown, one of the strictest restrictions in the COVID-19 pandemic. The findings of this study provide us with helpful knowledge to make appropriate considerations in case of future pandemic crises. 

A limitation of the current study is that we only gained insight in the experiences of parents during the COVID-19 pandemic and we were unable to compare experiences during the pandemic with experiences from parents in regular times. For future research, we would like to investigate parents’ experiences with newborn hearing screening in a time without COVID-19 restrictions. In addition, we would like to ask parents not only in retrospect about their experiences with newborn hearing screening, but also in advance about their beliefs and worries on hearing screening. Furthermore, it would be interesting to include experiences of hearing screeners in a future study as well. 

## Figures and Tables

**Table 1:** Participant characteristics.

Characteristic	Yes	No
	*n* (%)	*n* (%)
Postponed hearing screening	573 (54.4%)	480 (45.6%)
Born premature	49 (4.7%)	1004 (95.3%)
First child	469 (44.5%)	584 (55.5%)
Deaf/hard-of-hearing in the family	37 (3.5%)	1016 (96.5%)

**Table 2:** Statements on the hearing screening as reported by the parents.

	Statement	Mean (SD)	Disagree *n* (%)	Neutral *n* (%)	Agree *n* (%)
	Beliefs				
1	I recommend friends with babies to participate in the hearing screening	4.52 (0.74)	16 (1.5%)	73 (6.9%)	964 (91.6%)
2	The hearing screening unnecessarily worries parents	1.88 (0.83)	835 (79.3%)	184 (17.5%)	34 (3.2%)
	Experiences				
3	The screener showed interest in me and my baby	4.13 (0.91)	64 (6.1%)	129 (12.2%)	860 (81.7%)
4	The screener walked us through what he/she was doing during the hearing screening	4.36 (0.75)	30 (2.9%)	53 (5.0%)	970 (92.1%)
5	I felt at ease with the screener	4.27 (0.83)	42 (4.0%)	88 (8.4%)	923 (87.6%)
6	The hearing screening has upset my baby	1.48 (0.82)	951 (90.3%)	58 (5.5%)	44 (4.2%)
	Information				
7	I received enough information on the procedures of the hearing screening	4.10 (0.90)	78 (7.4%)	93 (8.8%)	882 (83.8%)
8	I received enough information beforehand to decide whether my baby had to be screened or not	3.38 (1.21)	282 (26.8%)	236 (22.4%)	535 (50.8%)
9	The procedure after the hearing screening has been clearly explained to me	3.52 (1.13)	208 (19.8%)	230 (21.8%)	615 (58.4%)
	Worries				
10	I felt relieved after the hearing screening of my baby	3.75 (0.87)	67 (6.4%)	319 (30.3%)	667 (63.3%)
11	Before the hearing screening took place, I was worried about the hearing of my baby	1.63 (0.87)	923 (87.7%)	79 (7.5%)	51 (4.8%)
12	I am currently worried about the hearing of my baby	1.28 (0.55)	1032 (98.0%)	11 (1.0%)	10 (1.0%)

**Table 3:** Mean and standard deviation per statement on the hearing screening as reported per three categories: postponed or not postponed hearing screening, prematurely or not prematurely born child, and first child or not first child.

	Statement	Postponed (*n* = 573)	Not Postponed (*n* = 480)	Premature (*n* = 49)	Not Premature (*n* = 1004)	First Child (*n* = 469)	Not First Child (*n* = 584)
	Information						
7	I think I received enough information on the procedures of the hearing screening	**4.04 (0.97)**	**4.17 (0.80)**	4.12 (0.97)	4.10 (0.90)	**4.04 (0.95)**	**4.15 (0.86)**
8	I think I received enough information beforehand to decide whether my baby had to be screened or not	3.32 (1.25)	3.44 (1.16)	3.29 (1.26)	3.38 (1.21)	**3.26 (1.20)**	**3.47 (1.20)**
9	The procedure after the hearing screening has been clearly explained to me	**3.39 (1.21)**	**3.69 (1.00)**	3.59 (1.04)	3.52 (1.13)	**3.38 (1.16)**	**3.64 (1.09)**
	Worries						
10	I felt relieved after the hearing screening of my baby	**3.68 (0.88)**	**3.83 (0.85)**	3.69 (1.05)	3.75 (0.86)	3.78 (0.88)	3.72 (0.86)
11	Before the hearing screening took place, I was worried about the hearing of my baby	1.65 (0.88)	1.61 (0.85)	**1.82 (1.07)**	**1.62 (0.85)**	1.69 (0.90)	1.58 (0.84)
12	I am currently worried about the hearing of my baby	1.24 (0.49)	1.32 (0.61)	1.41 (0.73)	1.27 (0.54)	1.29 (0.56)	1.27 (0.54)

Significant results are presented in bold.

## Data Availability

Data are not publicly available because we do not have consent in favor of public access of the parents of the subjects involved.
